# Multidimensional motivation for exercise: A latent profile and transition analysis

**DOI:** 10.1016/j.psychsport.2019.101619

**Published:** 2020-03

**Authors:** Lydia G. Emm-Collison, Simon J. Sebire, Ruth Salway, Janice L. Thompson, Russell Jago

**Affiliations:** 1 Centre for Exercise, Nutrition & Health Sciences, School for Policy Studies, University of Bristol, 8 Priory Road, Bristol, BS8 1TZ, UK; 2 School of Sport, Exercise and Rehabilitation Sciences, University of Birmingham, Birmingham, B15 2TT, UK

**Keywords:** Motivation, Physical activity, Latent profile, Latent transition, Accelerometer

## Abstract

**Objectives:**

To: a) identify motivational profiles for exercise, using Self-Determination Theory as a theoretical framework, among a sample of parents of UK primary school children; b) explore the movement between motivational profiles over a five year period; and c) examine differences across these profiles in terms of gender, physical activity and BMI.

**Design:**

Data were from the B-Proact1v cohort.

**Methods:**

2555 parents of British primary school children participated across three phases when the child was aged 5–6, 8–9, and 10–11. Parents completed a multidimensional measure of motivation for exercise and wore an ActiGraph GT3X + accelerometer for five days in each phase. Latent profile and transition analyses were conducted using a three-step approach in MPlus.

**Results:**

Six profiles were identified, comprising different combinations of motivation types. Between each timepoint, moving between profiles was more likely than remaining in the same one. People with a more autonomous profile at a previous timepoint were unlikely to move to more controlled or amotivated profiles. At all three timepoints, more autonomous profiles were associated with higher levels of MVPA and lower BMI.

**Conclusions:**

The results show that people’s motivation for exercise can be described in coherent and consistent profiles which are made up of multiple and simultaneous types of motivation. More autonomous motivation profiles were more enduring over time, indicating that promoting more autonomous motivational profiles may be central to facilitating longer-term physical activity engagement.

## Highlights

• Six qualitatively different profiles of motivation for exercise were identified.
• More autonomous profiles were associated with higher accelerometer estimated MVPA.
• Movement between profiles was dynamic and more autonomous profiles were more stable.
• Personal value and enjoyment of exercise appear to persist over time.

Regular physical activity is associated with a reduced risk of several health outcomes, including cardiovascular disease, stroke, type 2 diabetes, some forms of cancer, and depression (≥2020 counts per minute; Kyu et al., 2016; Rebar et al., 2015). To achieve these health benefits, adults aged 19–64 years are recommended to participate in at least 150 min of moderate-to-vigorous physical activity (MVPA), or 75 min of vigorous physical activity, every week (Canadian Society for Exercise Physiology, 2016; Chief Medical Officers, 2011; Department of Health, 2019; US Department of Health and Human Services, 2018). However, evidence indicates that as many as 43% of adults living in developed countries do not meet these recommendations, with men consistently engaging in more activity than women (Colley et al., 2011; Hallal et al., 2012; Kapteyn et al., 2018). Evidence also suggests an association between body weight and physical activity, with adults who have a higher body mass index (BMI) engaging in less physical activity than adults with a healthy BMI (Colley et al., 2011). It is widely acknowledged that public awareness of the physical activity guidelines is low (Kay, Carroll, Carlson, & Fulton, 2014; LeBlanc et al., 2015), however most adults are aware of the benefits of being physically active and therefore the low levels of activity may be indicative of low motivation and/or failures of self-regulatory processes (Lachman, Lipsitz, Lubben, Castaneda-Sceppa, & Jette, 2018). Parents of dependent-aged children make up a significant proportion of the UK adult population (Office for National Statistics, 2017) and parents of young children are less physically active than non-parents (Bellows-Riecken & Rhodes, 2008; Berge, Larson, Bauer, & Neumark-Sztainer, 2011). There is also evidence indicating that promoting more physical activity in parents could have benefits for them and their child, in terms of health and parenting behaviour (Hamilton & White, 2010). Understanding the processes that underpin adults, and specifically parents, physical activity, and consequently identifying routes through which to promote greater physical activity engagement, is a key public health objective.

Motivation is consistently shown to be related to physical activity (Choi, Lee, Lee, Kang, & Choi, 2017). Traditional theories conceptualise motivation as a dichotomous construct where individuals are either intrinsically or extrinsically motivated (Bandura, 1996), however such theories do not adequately reflect the complexity and multidimensionality of motivation. Self-determination theory (SDT) has gained significant attention in the sport and exercise psychology literature (Lindahl, Stenling, Lindwall, & Colliander, 2015) and offers a framework through which to investigate motivation quality (Ryan & Deci, 2017). Within SDT, quality of motivation is placed on a continuum whereby different types of motivation differ in the extent to which they are autonomous or controlled (Deci & Ryan, 2000; Howard, Gagne, & Bureau, 2017). Autonomous motivation is comprised of three types of behavioural regulation: intrinsic motivation, the most autonomous, is characterised by enjoyment and satisfaction of being active, integrated regulation is when the behaviour aligns with the person’s identity, and identified regulation is characterised by personal value and meaning attributed to being active (Ryan & Deci, 2017). Controlled motivation is also comprised of two types of behavioural regulation: introjected regulation is characterised by behaviour driven by internal pressures, such as being active to avoid feelings of guilt and shame and external regulation is where behaviour is driven by external pressures from other people (Deci & Ryan, 2000). In addition to these types of regulation, a lack of motivation is referred to as amotivation (Ryan & Deci, 2017).

In the context of physical activity and exercise behaviours, more autonomous motivation for exercise is consistently associated with higher self-reported and accelerometer-estimated physical activity (Solomon-Moore et al., 2016; Standage, Sebire, & Loney, 2008). Additionally, longitudinal evidence suggests that autonomous forms of motivation are associated with sustained physical activity engagement over periods ranging from 1-month to 5-years (Barbeau, Sweet, & Fortier, 2010; Emm-Collison, Jago, Salway, Thompson, & Sebire, 2019; Gunnell, Crocker, Mack, Wilson, & Zumbo, 2014). A recent meta-analysis of physical activity interventions indicates that promoting more autonomous motivation for exercise (e.g. through autonomy-supportive communication) is effective for facilitating long-term behaviour change (Samdal, Eide, Barth, Williams, & Meland, 2017). However, there is mixed evidence regarding the role of controlled motivation with most cross-sectional studies showing little association between this construct and physical activity (Duncan, Hall, Wilson, & Jenny, 2010; Solomon-Moore et al., 2016; Standage et al., 2008). In part, this is due to the aggregation of external and introjected regulation to a composite score of controlled motivation. In the few studies that have examined each behavioural regulation type separately, introjected regulation (i.e. being active to avoid feelings of guilt or shame) has been shown to have no cross-sectional association with accelerometer-assessed physical activity (Solomon-Moore et al., 2016; Standage et al., 2008). However, some longitudinal evidence suggests that high levels of introjected regulation may lead to a small decline in moderate-to-vigorous physical activity (MVPA) over time (Emm-Collison et al., 2019).

The majority of SDT-based evidence in physical activity context has taken a variable-centred approach, allowing inferences to be made regarding the independent associations between each type of behavioural regulation and behaviour. Both qualitative (Sebire et al., 2018) and quantitative (Lindwall et al., 2017) literature supports the layman perspective that people’s behaviours are motivated by multiple different reasons simultaneously and this aligns with theoretical assumptions (Howard, Gagne, Morin, & Van den Broeck, 2016; Lindwall et al., 2017; Vansteenkiste & Mouratidis, 2016). However, traditional variable-centred analysis (e.g. regression) does not account for this multidimensionality nor allow the exploration of the interplay between the different types of behavioural regulation and behavioural outcomes. For example, it is unknown whether multiple simultaneous motivations are beneficial for behaviour, as when one motivation source weakens there are others to fall back on (Cox & Ullrich-French, 2009), or whether experiencing high levels of controlled motivation alongside autonomous motivation has a detrimental influence on behaviour compared to being motivated for purely autonomous reasons (Boiche, Sarrazin, Grouzet, Pelletier, & Chanal, 2008).

There is an emerging body of evidence that has adopted a person-centred approach to analysis in the broader SDT literature (Howard et al., 2016; Jaakkola, Wang, Soini, & Liukkonen, 2015; Martinent & Decret, 2015) and in the physical activity context, using both cluster-analysis (Guerin & Fortier, 2012; Matsumoto & Takenaka, 2004) and, more recently, latent profile analysis (Bechter, Dimmock, Howard, Whipp, & Jackson, 2018; Lindwall et al., 2017). These studies have identified several profiles that combine different levels of each type of behavioural regulation, resulting in several distinct combinations of motivation. This provides support for the multi-dimensional nature of motivation and highlights that variable-centred approaches are not able to fully account for this multidimensionality. Across studies, there has been some consistency in profiles, particularly those characterised by high levels of autonomous motivation and, at the other end of the motivational continuum, those characterised by high levels of amotivation (Bechter et al., 2018; Lindwall et al., 2017). However, there has been less consistency in the less extreme profiles. Some studies have offered additional evidence for the validity of the profiles, by investigating the theoretical pre-cursors of motivation. For example, Lindwall et al. (2017) found that self-determined motivational profiles (i.e. those characterised by high levels of intrinsic and identified regulation) were associated with satisfaction of needs for autonomy, competence and relatedness as predicted in SDT. There is also some evidence indicating that more self-determined profiles are associated with higher levels of physical activity (Lindwall et al., 2017). However, this evidence is based on self-report measures of activity, which have been shown to provide non-systematically unreliable estimates of physical activity (Prince et al., 2008), and to date no study has related motivational profiles to objectively-estimated physical activity. Further, there has been no exploration of motivational profiles in the context of longitudinal data, and therefore the stability of such profiles is unknown. Such analyses would allow for the exploration of the relative stability of profile membership (Vansteenkiste & Mouratidis, 2016) and, in doing so would help to identify individuals who may be at risk of becoming physically inactive due to likely changes in their motivation profile.

Existing evidence indicates that there are no gender differences in motivation for physical activity (see Guerin, Bales, Sweet, and Fortier (2012) for a review). However, this review focuses on mean levels of the different behavioural regulation types and gender differences may not manifest in this way (Guerin et al., 2012). More recent research has found differences in the way in which behavioural regulations are associated with exercise behaviour, for example introjected regulation was found to be positively associated with exercise in men but negatively associated with exercise in women (Weman-Josefsson, Lindwall, & Ivarsson, 2015). Such differences in association may be explained by gender differences in the way behavioural regulations combine and thus it may be that men and women are more likely to have different profiles of motivation. From the perspective of designing targeted interventions, it is therefore important to explore the associations between profile membership and gender. There may also be associations between motivation for physical activity and body mass index with different levels of behavioural regulations between weight categories (Hwang & Kim, 2013; Mokhtari et al., 2017). Further, there appears to be associations between motivation for physical activity and other weight control behaviours such as eating self-regulation, which has been termed ‘motivational spill-over’ (Mata et al., 2009).

## 1 Aims

The purpose of the present exploratory study was to adopt a person-centred approach to: a) identify motivational profiles for exercise amongst adults, using Self-Determination Theory (SDT) as a theoretical framework; b) explore the stability of and movement between motivational profiles over a five-year period; and c) examine differences across these profiles in terms of gender, accelerometer-estimated physical activity and BMI.

## 2 Methods

### 2.1 Design and participants

This study uses data from the longitudinal B-Proact1v cohort study. A more detailed outline of the study can be found elsewhere (Jago et al., 2017, Jago et al., 2019, Jago et al., 2014, Jago et al., 2014). In brief, B-Proact1v aimed to examine physical activity and sedentary behaviour of primary school children and their parents. Data were collected on three occasions, between January 2012 and July 2013 when the child was in Year 1 (ages 5–6), between March 2015 and July 2016 when the same child was in Year 4 (ages 8–9), and between March 2017 and May 2018 when the same child was in Year 6 (ages 10–11). A total of 57 schools participated in the first data collection, and the same schools were invited to take part in subsequent phases, with 47 participating in the second phase and 50 in the third phase. Across the three timepoints, data were collected from 2555 parents/caregivers from 2132 families: 1195 were involved at time 1, 1140 at time 2, and 1233 at time 3. Prior to data collection, the study received ethical approval from the School for Policy Studies Research Ethics Committee at the University of Bristol and written consent was obtained from all participants at each phase of data collection.

### 2.2 Measures

#### 2.2.1 Exercise motivation

Motivation to exercise was measured via the Behavioural Regulation in Exercise Questionnaire-2 (BREQ-2; Markland & Tobin, 2004). Grounded in SDT, the 19-item measure assesses five forms of behavioural regulation; intrinsic (4 items e.g. ‘*I exercise because it’s fun’*), identified (4 items e.g. *‘It’s important to me to exercise regularly’*), introjected (3 items e.g. *‘I feel like a failure when I haven’t exercise in a while’*), external (4 items e.g. *‘I exercise because other people say I should’*), and amotivation (4 items e.g. *‘I don’t see the point in exercising’*). Participants recorded their responses on a 5-point Likert scale ranging from 0 (*not true for me*) to 4 (*very true for me*). The subscales demonstrated internal consistency at each time point (Table 1).

**Table 1:** Descriptive statistics for all study variables at each timepoint.

*Variable*	Time 1 (N = 1023)			Time 2 (N = 925)			Time 3 (N = 891)		
	Mean (SD)	% missing	α	Mean (SD)	% missing	α	Mean (SD)	% missing	α
Gender (% female)	76.3%	–	–	72.6%	–	–	73.0%	–	–
Age (years)	37.77 (5.72)	–	–	41.34 (6.27)	–	–	43.03 (5.99)	–	–
BMI	25.40 (4.54)	–	–	25.92 (25.86)	–	–	25.86 (4.80)	–	–
MVPA	49.54 (24.24)	–	–	50.44 (25.11)	–	–	51.86 (25.61)	–	–
Intrinsic motivation	2.56 (1.01)	–	0.92	2.50 (1.12)	.04%	0.93	2.50 (1.12)	.06%	0.93
Identified regulation	2.64 (0.99)	–	0.83	2.63 (0.97)	.04%	0.85	2.63 (0.96)	.08%	0.85
Introjected regulation	1.26 (1.01)	–	0.75	1.36 (1.05)	.04%	0.78	1.31 (1.06)	.06%	0.79
External regulation	0.28 (0.50)	–	0.70	0.38 (0.61)	.05%	0.78	0.33 (0.55)	.08%	0.75
Amotivation	0.24 (0.54)	–	0.68	0.27 (0.56)	.06%	0.72	0.26 (0.55)	.06%	0.74

#### 2.2.2 Moderate-to-vigorous physical activity

Parents wore an ActiGraph wGT3X-BT accelerometer on their waist for five days, including two weekend days. Accelerometer data were processed using Kinesoft software (v3.3.75; Kinesoft, Saskatchewan, Canada) using 60-s epochs. A valid day was defined as at least 500 min of data after the exclusion of periods of non-wear time of over 60 min, whilst allowing up to 2 min of interruptions. Analysis was restricted to those parents who provided at least three days of valid data, to ensure reasonable estimates of typical daily activity whilst maximising sample size (Aadland & Ylvisaker, 2015; Tudor-Locke et al., 2005). The average number of MVPA minutes per day were used in the analysis, derived for each participant using population-specific cut points for adults (≥2020 counts per minute; Troiano et al., 2008).

#### 2.2.3 Participant characteristics

Parents reported their date of birth and gender. BMI was calculated from self-reported height and weight as weight (kg)/height (m^2^).

### 2.3 Data analysis

First, confirmatory factor analysis using maximum likelihood estimation was conducted to obtain weighted factor-scores for each subscale of the BREQ-2 measure (intrinsic motivation, identified regulation, introjected regulation, extrinsic regulation, and amotivation). Doing so provides subscale estimates that consider the contribution of each item to the latent variable they are measuring. Model fit was assessed using multiple indices as follows; the Chi-square index, comparative fit index (CFI), standardised root mean square residual (SRMR), and root mean square of approximation (RMSEA). The thresholds for good fit used were >0.90 for the CFI, <0.08 for the SRMR, and <0.06 for the RMSEA (Hu & Bentler, 1999). Longitudinal invariance of the measurement model was sequentially tested via a series of increasingly constrained models. Invariance was indicated by a change in CFI of ≤0.01 (Cheung & Rensvold, 2002). The generated factor scores were used as input variables in subsequent analyses, in line with guidance (DiStefano, Zhu, & Mindrila, 2009). We used latent profile analysis (LPA) as the primary data analysis approach to explore and identify motivational profiles for exercise. In LPA, each participant is assumed to belong to one of a set of underlying profiles, and the analysis estimates the probability of membership to each profile for each person. As an extension of LPA, latent transition analysis was used to additionally estimate the probability of moving between profiles at different time points.

We used a three-step approach (Asparouhov & Muthen, 2014; Nylund-Gibson, Grimm, Quirk, & Furlong, 2014) to conduct the LPA and transition analyses in MPlus (version 7, Muthen & Muthen). In a fourth step we explored the associations of profile membership with gender, BMI and MVPA. The syntax for all main analyses is available as supplementary material.

#### 2.3.1 Step 1 and 2- identification of latent profiles and obtaining classification errors

Through step 1 we identified the motivational profiles for each timepoint. A sequence of models, with an increasing number of profiles from 2 to 7, were examined to ascertain whether more complex (i.e. more profiles) or parsimonious (i.e. fewer profiles) models provided the best description of the data. The models were estimated using data from all three timepoints, with each time point assumed to be independent of the others. Based on recommendations (Nylund, Asparoutiov, & Muthen, 2007), and in line with previous papers (Jago et al., 2018; Lindwall et al., 2017), several criteria were used to determine the most appropriate model. Statistically, the log-likelihood, the Bayesian information criterion (BIC) and the sample-adjusted Bayesian information criterion (SSA-BIC) were considered, with lower values indicating better model fit (Henson, Reise, & Kim, 2007; Yang, 2006). Relative entropy and the class membership probabilities were used to identify potentially problematic models in which some classes have small proportions of membership. We also considered the theoretical alignment and interpretation of the final profiles in terms of different levels of behavioural regulation (Vansteenkiste & Mouratidis, 2016) and, with this in mind, sought to choose the most meaningful model with the smallest number of profiles. In order to explore assumptions about variance, we compared the model with no constraints, correlated indicators and equal variances across timepoints (Morin, Meyer, Creusier, & Bietry, 2016).

In the second step, to include the measurement error in assignment of individuals to profile, we conducted latent profile analysis separately for each set of latent profile indicators, fixing the measurement parameters so that the profiles were the same as in step 1. This allowed us to obtain profile variables and classification errors. This step was repeated for all three timepoints.

#### 2.3.2 Step 3- Transition Between Profiles Across timepoints

In the third step, we estimated the movement between motivation profiles across the three timepoints, keeping the latent profiles at each timepoint fixed and accounting for measurement error in profile assignment.

#### 2.3.3 Step 4- associations of profile membership with gender, BMI and MVPA

We examined the associations between profile membership and gender, BMI, and accelerometer-estimated MVPA, via the Wald test using the BCH method, which includes classification error and is robust to violations of assumptions (Yang, 2006).

All analyses accounted for clustering of parents at the family and school levels. Between timepoints, attrition was largely attributed to school drop-out, accounting for the drop out of 244 parents at time 2 and 167 parents at time 3, or to families moving to schools not involved in the project, accounting for a total of 253 parents. Therefore, as most missing data was explained by school-level rather than individual-level factors, all model parameters were calculated using full information maximum likelihood, which uses available information from participants at all time points and handles missing data within the analysis model, under the assumption that data are missing at random. Models were estimated using multiple start values (500 starts and 100 sets) to check convergence.

## 3 Results

### 3.1 Preliminary analysis

1023 participants provided valid accelerometer measurements at time 1 (86% of those in the study at time 1), 925 at time 2 (81% of those in the study at time 2), and 891 at time 3 (72% of those in the study at time 3). Table 1 shows descriptive statistics and proportions of missing values for questionnaire measures in these participants. Correlations between variables are presented in supplementary material (Table S1). At each timepoint, most participants were female (72–76%) and mean BMI was between 25 and 26 kg/m^2^. At time 1, the mean age was 37.8 years increasing to 41.3 at time 2 and 43.0 at time 3. The average daily minutes of MVPA increased across timepoints, from 49.5 min per day at time 1 to 50.4 min per day at time 2 and 51.86 min per day at time 3. At all three timepoints, the sample had similar motivational distributions, with low levels of amotivation and high levels of both identified regulation and intrinsic motivation. Factors scores from BREQ-2 were derived via CFA.^1^ The model showed acceptable fit to the data at time 1 (χ^2^ = 574.38, df = 142, p < .0005; CFI = 0.93, RMSEA = 0.05 (90% CI = 0.05, 0.06), SRMR = 0.05), time 2 (χ^2^ = 496.66, df = 142, p < .0005; CFI = 0.95, RMSEA = 0.05 (90% CI = 0.05, 0.06), SRMR = 0.05), and time 3 (χ^2^ = 418.44, df = 142, p < .0005; CFI = 0.96, RMSEA = 0.04 (90% CI = 0.04, 0.05), SRMR = 0.04). Factor determinacy scores ranged from 0.86 to 0.97 and were deemed to provide a good estimate of the true factor score (Table S2). The results of the invariance testing provided evidence for the equivalence of the measurement model across timepoints (Table S3). Prior to running the main analyses, behavioural regulation variables were checked for univariate and multivariate outliers, and no meaningful outliers were detected (Tabachnick & Fidell, 2013).

#### 3.1.1 Step 1 and 2- identification of latent profiles and obtaining classification errors

Table 2 shows the indicators of model fit for latent profile models containing 2–7 profiles. The log-likelihood decreased as the number of profiles increased and the 6-profile model had the lowest BIC indicating a better fit than the models containing fewer profiles. Whilst the 7-profile solution had a lower SSA-BIC, at each timepoint at least one profile had a very low probability of membership (<2%). The 6-profile solution had the next lowest SAA-BIC and all profiles had reasonable membership probabilities (over 4%). Compared to the 7-profile solution, the 6-profiles were more meaningful, with combinations of different types of behavioural regulation representing logical and theoretically-appropriate profiles. Additionally, we ran the model with alternative specifications (1000 starts and 200 sets) and the log-likelihood was replicated. We therefore chose to proceed with 6 profiles as the most appropriate model, based on a combination of log-likelihood, BIC, SSA-BIC and model interpretability. With regards to measurement invariance (Morin et al., 2016), models with different constraints produced similar results (Tables S5 and S6), and so, for parsimony and given that similar patterns of motivation exist across populations and contexts (Milyavskaya & Koestner, 2011), we proceeded with the model that assumed that the variances for each motivation variable differed between profiles but were equal across timepoints.

**Table 2:** Fit indices and model comparisons for estimated latent profile analysis models.

*N. Classes*	Log-likelihood	BIC	SSA-BIC	Entropy
2 classes	−14052.81	28316.32	28227.36	.52
3 classes	−12815.78	25902.47	25788.10	.57
4 classes	−12208.00	24747.11	24607.32	.60
5 classes	−11639.102	23677.04	23508.66	.62
6 classes	−11192.90	22837.30	22646.68	.62
7 classes	−9730.04	23659.08	21886.33	.62

*Note*: BIC Bayesian Information Criterion, SSA-BIC Sample size adjusted Bayesian information

**Figure 1:**
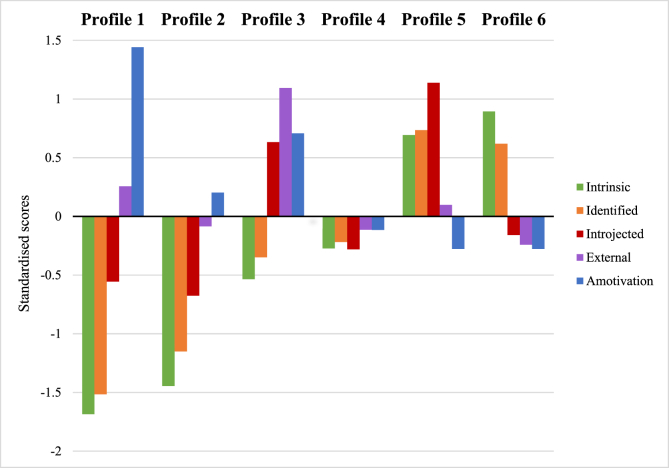
Motivational profiles for the six-profile model

**Figure 2:**
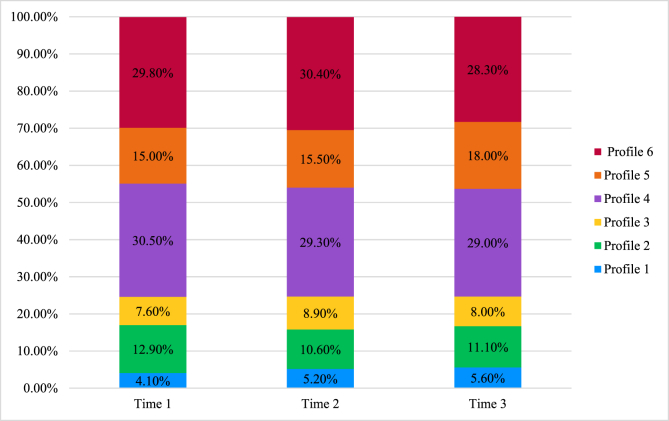
Probability of Membership to Each Profile at Each Timepoint

Details of the six profiles are reported in Table 3, the profiles are represented graphically in Figure 1, and the probability of membership to each profile is presented in Figure 2. To ensure that interpretation is theoretically meaningful and appropriate, we have re-ordered the profiles to match the motivational continuum proposed in SDT (i.e. from the least to the most self-determined). The six profiles were labelled as:

1. **Strongly amotivated-** Primarily amotivation with some external regulation
2. **Amotivated-** Moderate levels of amotivation with low levels of all other types of regulation
3. **Controlled and amotivated-** High levels of both introjected and external regulation accompanied by high levels of amotivation
4. **Low in motivation-**low levels of all types of behavioural regulation
5. **Autonomously motivated and introjected-** Predominantly introjected regulation alongside some intrinsic and identified regulation
6. **Autonomously motivated-** Primarily intrinsic motivation accompanied with identified regulation.

**Table 3:** Description of the six latent profiles based on standardised BREQ-2 variables across all timepoints and proportions of membership.

*Variable*	Profile 1 (Strongly amotivated)	Profile 2 (Amotivated)	Profile 3 (Controlled and amotivated)	Profile 4 (Low in motivation)	Profile 5 (Autonomously motivated and introjected)	Profile 6 (Autonomously motivated)
1. Intrinsic	−0.11	−1.32	−0.77	−0.16	0.92	−1.75
2. Identified	0.12	−1.05	−0.66	−0.12	0.71	−1.60
3. Introjected	0.83	−0.61	0.36	−0.25	0.27	−0.70
4. External	0.92	−0.09	1.12	−0.16	−0.19	0.09
5. Amotivation	0.08	0.17	1.14	−0.15	−0.28	1.41

*Note*. Positive values represent a strong endorsement of the behavioural regulation type and negative values indicate a weak endorsement of the behaviour regulation type. Values closer to zero indicate neutral responses.

At all three timepoints, a greater proportion of participants were likely to be assigned to Profile 3 (low in motivation) or Profile 6 (autonomously motivated). Participants were consistently least likely to belong to Profile 1 (strongly amotivated; see Figure 2).

#### 3.1.2 Comparison of profiles at time 1, 2 and 3

Figure 2 illustrates the probabilities of profile membership at each timepoint. Proportions of participants in each profile were similar over time, with Profile 6 (autonomously motivated) and 4 (low in motivation) being the largest. At each time point, Profile 1 (strongly amotivated) had the lowest membership. The proportion of participants in Profile 5 (autonomously motivated with introjected) increased across timepoints, particularly across time 2 and 3.

#### 3.1.3 Step 3- Transition Between Profiles Across timepoints

**Figure 3:**
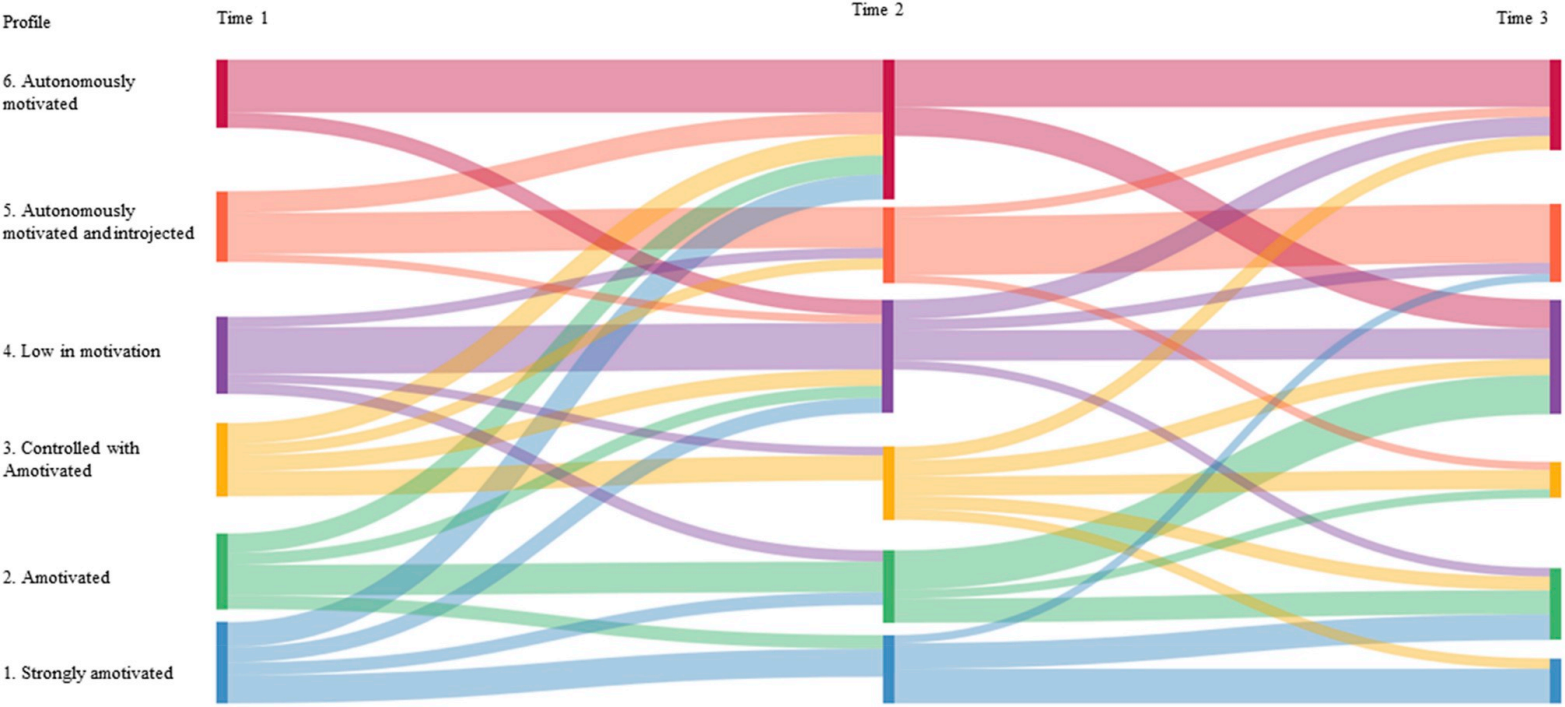
Transition between profiles across the three timepoints

The likely patterns of movement between profiles across the three timepoints are shown in Figure 3. Between time 1 and 2, a large proportion of participants were likely to remain in the same profile (47%). Participants who transitioned between profiles (53%) were likely to move to more motivationally-positive profiles, with participants having the highest probability of moving to profiles that were more autonomous. The exception to this was those in Profile 4 (low in motivation) being equally likely to move to Profiles 5 and 6 (both characterised by strongly autonomous regulations) or Profile 3 (controlled and amotivation). If those in Profile 6 (autonomously motivated) at time 1 were to move (19%) they were most likely to move to Profile 4 (low in motivation).

Between time 2 and 3, a similar proportion of participants were likely to remain in the same profile (45%). The probability of movement between profiles across time 2 and 3 was more varied, with a greater likelihood of moving to a wider range of profiles. In line with the stability found across time 1 and 2, those in Profiles 5 and 6 (both with high levels of autonomous motivation) and Profile 1 (highly amotivated) at time 2 were most likely to remain in the same profile at time 3. Those participants who were likely to move from Profiles 5 and 6 had the highest probability of moving to Profile 4 (low in motivation). Those who moved from profile 1 (high amotivation) were most likely to move to Profile 2 (amotivated), with very few moving to more self-determined profiles. For Profiles 3 (controlled and amotivated) and 4 (low in motivation) movement was more diverse, with a relatively balanced probability of moving to profiles characterised by more autonomous motivation or those characterised by more amotivation.

#### 3.1.4 Step 4- associations of profile membership with gender, BMI and MVPA

Table 4 shows the associations of profile membership with co-variates at each timepoint. Across all timepoints, the proportion of female membership in a profile ranged from 66% to 87%. There were no consistent patterns in profile membership across each timepoint in terms of gender, but at both time 1 and time 2, Profiles 1 and 2 (strongly amotivated and amotivated respectively) had the highest proportions of female participants. At time 3, Profile 3 (controlled and amotivated) had the highest proportion of female participants. BMI ranged from 24 to 28 across profiles and timepoints. Those in Profile 6 (autonomously motivated) had the lowest mean BMI at each timepoint and, generally, those in Profiles 1 and 3 (strongly amotivated and controlled and amotivated) had the highest BMI. There was less consistency in the association between profile and MVPA across the three timepoints. However, at each timepoint, there was a 15–17 min variation in MVPA across profiles. Profile 6 (autonomously motivated) was consistently associated with higher MVPA, and Profile 1 (Strongly amotivated) consistently associated with the lowest MVPA. At each timepoint, participants in Profile 5 (autonomously motivated and introjected) engaged in less MVPA than participants in Profile 6. For all timepoints, profiles with higher MVPA also had a lower BMI indicating an association between these variables.

**Table 4:** Differences across the six latent profiles in terms of gender, BMI and MVPA at all three timepoints.

*Variable*	Profile 1 (Strongly amotivated)	Profile 2 (Amotivated)	Profile 3 (Controlled and amotivated)	Profile 4 (Low in motivation)	Profile 5 (Autonomously motivated and introjected)	Profile 6 (Autonomously motivated)	
	M (S.E.)	M (S.E.)	M (S.E.)	M (S.E.)	M (S.E.)	M (S.E)	*p*
Time 1							
Gender (Prob female)	0.82 (0.06)	0.82 (0.03)	0.76 (0.05)	0.79 (0.02)	0.77 (0.04)	0.70 (0.03)	.08
BMI	27.71 (0.84)	25.43 (0.38)	26.69 (0.57)	26.29 (0.30)	25.20 (0.34)	24.02 (0.21)	.00
MVPA	38.77 (2.52)	42.20 (1.52)	49.44 (2.42)	45.20 (1.07)	54.02 (1.97)	56.27 (1.48)	.00
Time 2							
Gender (Prob female)	0.87 (0.05)	0.78 (0.04)	0.69 (0.05)	0.75 (0.03)	0.66 (0.05)	0.71 (0.03)	.02
BMI	28.57 (0.86)	27.41 (0.56)	28.68 (0.67)	25.99 (0.27)	25.70 (0.36)	24.23 (0.21)	.00
MVPA	41.46 (2.59)	49.39 (2.26)	47.17 (2.29)	44.49 (1.17)	55.25 (2.05)	56.54 (1.50)	.00
Time 3							
Gender (Prob female)	0.77 (0.06)	0.76 (0.04)	0.80 (0.05)	0.69 (0.03)	0.74 (0.04)	0.72 (0.03)	.45
BMI	28.21 (0.80)	27.22 (27.96)	27.96 (0.62)	26.72 (0.29)	25.35 (0.31)	23.81 (0.20)	.00
MVPA	42.58 (2.44)	51.86 (2.22)	45.50 (2.19)	49.52 (1.26)	51.35 (1.66)	58.49 (1.63)	.00

*Note*: *p*-value refers to the Wald test of differences between profiles.

## 4 Discussion

The evidence presented in this paper indicates that people have multiple simultaneous motivations for engaging in physical activity, providing further support for the complex multi-dimensional nature of physical activity behaviour. We identified six distinct motivational profiles that represented different combinations of motivation types spread across the continuum of motivation proposed within SDT. Further, whilst exploratory, this paper provides the first evidence for the movement of people between profiles over time, and the findings suggest that motivation is dynamic, with most participants moving between profiles across timepoints. Profiles consisting of strong endorsement of more autonomous forms of motivation were the most stable.

The six profiles identified were qualitatively different and distinct. Two profiles were characterised predominantly by a lack of motivation to exercise (amotivation), with participants in Profiles 1 and 2 having high and moderate levels of amotivation respectively. Profile 3 (controlled and amotivated) consisted of high levels of external regulation alongside amotivation and introjected regulation, indicating that a lack of interest in exercise, pressure from others and guilt and shame about not engaging in exercise do occur simultaneously. Participants in Profiles 5 and 6 reported moderate to high levels of both intrinsic and identified regulation, characterised by enjoyment and personal value of exercise, but Profile 5 had additional high levels of introjection. The structure of the profiles followed a logical progression along the theoretical motivation continuum, with combinations of closely-related regulation types indicating that similar types of motivation are more strongly correlated than disparate regulation types (Ryan & Deci, 2000). However, one profile did not align with the theoretical propositions within SDT, and that is Profile 4 (low in motivation), characterised by no distinct regulation type. A similar profile has been seen in previous papers (Lindwall et al., 2017) despite the theoretical expectation that low levels of both controlled and autonomous regulation types would be complimented with high levels of amotivation. This profile is therefore difficult to explain, and may be the result of response category artefact, where this profile comprises individuals who provided mid-range responses on the Likert scale (Nadler, Weston, & Voyles, 2015). Alternatively, given that the BREQ-2 questions refer specifically to exercise, this profile may represent a group who did not find the questions relevant due to not engaging in exercise (known as ‘exercise aschematic’; Kendzierski, 1990). In this sample, this profile represents a substantial group of participants (up to 30%) and so qualitative interviews with individuals likely to be classified to this profile could help to provide clarity on the source of this profile.

Several of the theoretically-meaningful profiles identified in this paper align with those found in previous profile analyses (Bechter et al., 2018; Lindwall et al., 2017). Specifically, Profile 6 (autonomously motivated), Profile 5 (autonomously motivated and introjection), Profile 1 (strongly amotivated), and Profile 4 (low in motivation) replicate profiles previously identified in both active and non-active adult populations (Lindwall et al., 2017). There have also been similar profiles identified in research with young people (Bechter et al., 2018). Collectively, this provides further confirmation of the validity of the profiles and indicates that similar combinations of behavioural regulations are observed across different samples, countries, and age-groups, providing further support for the universal nature of motivation as conceptualised in SDT.

The patterns of MVPA for each profile provide support for the construct validity of the profiles and the continuum of motivation proposed within SDT (Ryan & Deci, 2017). At all three timepoints, individuals most likely to be in Profile 6 (autonomously motivated) engaged in more MVPA than those in any other profile, with those in Profile 1 (strongly amotivated) consistently engaging in the least. Further, the difference in MVPA between the motivation profiles provides further support for person-centred analysis as a method to provide additional insight into the role of each regulation type and the way in which they may combine to influence behaviour. In particular, the profiles identified through this analysis indicate that introjection may not occur in high levels in isolation, but rather in two qualitatively different profiles, combined either with external regulation (as in Profile 3) or with identified and intrinsic regulation (as in Profile 5). Most of the SDT literature has found little association between introjected regulation and physical activity (Duncan et al., 2010; Standage et al., 2008), but the different combinations of behavioural regulation may mean that traditional analysis methods have masked the differential influence that introjected regulation can have, depending on which other behavioural regulations it occurs alongside. Further, a similar profile characterised by high self-determined and introjected regulation was found in previous studies associating motivation profiles with self-reported exercise behaviour (Lindwall et al., 2017), but this evidence indicated that introjected regulation did not have a detrimental influence on physical activity when found in combination with autonomous motivation. In contrast to this, our findings suggest that the presence of introjected regulation alongside autonomous motivation is associated with lower levels of MVPA compared to when autonomous motivation is experienced alone. Collectively, the evidence suggests that the addition of introjected regulation to an otherwise autonomously motivated person will, at best, have no impact or, at worst, undermine behaviour. Further work is needed to clarify the role of introjected regulation in determining physical activity behaviour.

The data presented here show that the autonomously motivated profile was associated with a lower BMI at each timepoint. This may be linked to the association between more autonomous motivation and higher levels of MVPA but may also represent a wider association between autonomous motivation for exercise and other weight control behaviours such as diet, sometimes referred to as ‘motivational spill-over’ (Mata et al., 2009). Additionally, at all three timepoints, participants in Profile 1 (strongly amotivated) had a high average BMI. This is consistent with previous research with adolescents and adults showing that individuals with a higher BMI are more likely to report high levels of amotivation and individuals who are have a healthy BMI are more likely to report intrinsic regulation for exercise (Ersoz, Altiparmak, & Asci, 2016; Hwang & Kim, 2013). The data therefore highlight a need for further examination of the associations between motivation, physical activity and body weight.

In the current sample, across all timepoints individuals were more likely to move between profiles than to remain in the same one, indicating that motivation for exercise is relatively dynamic. More autonomous profiles were the most stable across timepoints and participants likely to belong to profiles characterised by strong controlled motivation or amotivation were most likely to move to other profiles. This movement was most commonly to more autonomous profiles, which is consistent with the principle of SDT that humans have an innate desire to seek out situations and environments that are psychologically fulfilling and, over time and given satisfaction of autonomy, competence and relatedness needs, will become more self-determined in their motivation (Deci & Ryan, 2000). The findings also suggest that individuals with low levels of motivation may be motivationally vulnerable, in that over time they have a similar probability of moving to more autonomous or more amotivated profiles. This presents a potentially important opportunity to intervene to attempt to inspire people towards more stable autonomous forms of motivation.

From a public health perspective, these findings suggest that strategies to promote greater physical activity engagement should seek to foster more stable autonomous motivation by developing physical activity environments that support, rather than thwart, the basic psychology needs of autonomy, competence and relatedness (Ryan & Deci, 2017). The stability of the profiles identified in this paper indicate that doing so could have longer-term benefits for physical activity behaviour. Environments that provide choice about when and how to be active, opportunities for success, skill building, and an optimal level of challenge, a personally-relevant rationale for being active, and the opportunity to develop strong connections with others have been shown to promote greater physical activity engagement in the long-term (Samdal et al., 2017). There are several opportunities for future research with a clear need for more person-centred analyses of exercise motivation. As this is the first exploration of the transition between profiles, further research is needed to identify whether the stability of and movement between profiles is consistent across samples. Additionally, further work is needed to ascertain the reasons why individuals may move between motivational profiles and so the longitudinal assessment of wider theoretical constructs, such as need satisfaction and well-being, is needed. Qualitative work allow the origins of the low motivation profile to be explored, specifically focusing on whether this profile represents a genuine group of individuals, is the result of the measure used, or if it is an amalgamation of individuals with different motivational profiles.

### 4.1 Strengths and limitations

The exploratory person-centred analysis, longitudinal data and objective estimates of physical activity are particular strengths of this study, allowing the investigation of the interplay between different types of motivation and associations with physical activity, as well as exploring the stability of motivation over time. However, it is important to highlight the limitations of this work. First, the sample was largely female and therefore the profiles may be more representative of those found amongst women rather than men. Additionally, the sample were all parents of primary-school aged children and were mostly mothers, therefore may not represent the wider adult population. However, given that previous studies have found no gender differences in behavioural regulations (Guerin et al., 2012), and given the universality assumption of SDT, we would not anticipate major differences in the profiles we identified in a more balanced sample. There may, however, be differences in the proportions of parents belonging to particular profiles when compared to the wider population (McIntyre & Rhodes, 2009; Solomon-Moore et al., 2016). Additionally, our sample were high in autonomous forms of motivation, low in amotivation and generally active, which limits the generalisability of the profiles to groups with lower autonomous motivation and activity levels. The nature of the study in which this analysis was nested is likely to have influenced this sample, attracting parents who enjoy and value being active themselves, and so future work should aim to recruit a more representative and varied sample. Further, whilst we assumed that data were missing at random, it is possible that parents who provided full data may have higher quality motivation than those with missing data. It is also to be noted that BMI was based on self-reported indicators and therefore may not be accurate. The consistent associations between BMI and motivation profiles across the three timepoints provide strong support for this relationship, but future research should adopt objective measurements of height and weight to provide additional clarity. Additionally, whilst we did not have sufficiently powered sample to do so, future work should seek to explore the associations between profile transition and MVPA and BMI and additional covariates, such as need satisfaction and need frustration.

## 5 Conclusion

This paper provides evidence for the experience of multiple simultaneous reasons for engaging in exercise and that more autonomous motivation profiles are associated with higher levels of accelerometer assessed MVPA and lower BMI. The latent transition analysis provides the first evidence that profiles characterised by autonomous forms of motivation are more stable over time than less self-determined profiles. This indicates that once individuals establish and personal value of enjoyment of exercise, this persists over time, and so promoting more autonomous motivational profiles may be central to facilitating long-term physical activity engagement.

## Declaration of competing interest

We have no competing interests to report.

## Footnotes

1 An exploratory structural equation model was also run to obtain factor scores, the details of which can be found in Table S4. However, as there were not substantial differences in model fit between the CFA and ESEM, subsequent analyses proceed with factor scores generated from the CFA.

## References

[bib1] Aadland E., Ylvisaker E. (2015). Reliability of the actigraph GT3X+Accelerometer in adults under free-living conditions. PLoS One.

[bib2] Asparouhov T., Muthen B. (2014). Auxiliary variables in mixture modeling: Three-step approaches using mplus. Structural Equation Modeling-a Multidisciplinary Journal.

[bib3] Bandura A. (1996). Failures in self-regulation: Energy depletion or selective disengagement?. Psychological Inquiry.

[bib4] Barbeau A., Sweet S.N., Fortier M. (2010). A path-analytic model of self-determination theory in a physical activity context. Journal of Applied Biobehavioral Research.

[bib5] Bechter B.E., Dimmock J.A., Howard J.L., Whipp P.R., Jackson B. (2018). Student motivation in high school physical education: A latent profile Analysis approach. Journal of Sport & Exercise Psychology.

[bib6] Bellows-Riecken K.H., Rhodes R.E. (2008). A birth of inactivity? A review of physical activity and parenthood. Preventive Medicine.

[bib7] Berge J.M., Larson N., Bauer K.W., Neumark-Sztainer D. (2011). Are parents of young children practicing healthy nutrition and physical activity behaviors?. Pediatrics.

[bib8] Boiche J.C.S., Sarrazin P.G., Grouzet F.M.E., Pelletier L.G., Chanal J.P. (2008). Students' motivational profiles and achievement outcomes in physical education: A self-determination perspective. Journal of Educational Psychology.

[bib9] Canadian Society for Exercise Physiology (2016). Canadian physical activity guidelines.

[bib10] Cheung G.W., Rensvold R.B. (2002). Evaluating goodness-of-fit indexes for testing measurement invariance. Structural Equation Modeling-a Multidisciplinary Journal.

[bib11] Chief Medical Officers (2011). Start active, stay active: A report on physical activity from the four home countries.

[bib12] Choi J., Lee M., Lee J.K., Kang D., Choi J.Y. (2017). Correlates associated with participation in physical activity among adults: A systematic review of reviews and update. BMC Public Health.

[bib13] Colley R.C., Garriguet D., Janssen I., Craig C.L., Clarke J., Tremblay M.S. (2011). Physical activity of Canadian adults: Accelerometer results from the 2007 to 2009 Canadian health measures survey. Health Reports.

[bib14] Cox A.E., Ullrich-French S. (2009). Examining combinations of peer and teacher relationship variables in physical education. Journal of Sport & Exercise Psychology.

[bib15] Deci E.L., Ryan R.M. (2000). The "what" and "why" of goal pursuits: Human needs and the self-determination of behavior. Psychological Inquiry.

[bib16] Department of Health (2019). Australia's physical activity & sedentary behavior guidelines for adults (18-64 years).

[bib70] DiStefano C., Zhu M., Mindrila D. (2009). Understanding and Using Factor Scores: Considerations for the Applied Researcher. Practical Assessment, Research & Evaluation.

[bib17] Duncan L.R., Hall C.R., Wilson P.M., Jenny O. (2010). Exercise motivation: A cross-sectional analysis examining its relationships with frequency, intensity, and duration of exercise. International Journal of Behavioral Nutrition and Physical Activity.

[bib18] Emm-Collison L., Jago R., Salway R., Thompson J.L., Sebire S.J. (2019). Longitudinal associations between parents' motivation to exercise and their moderate-to-vigorous physical activity. Psychology of Sport and Exercise.

[bib19] Ersoz G., Altiparmak E., Asci F.H. (2016). Does body mass index influence behavioral regulations, dispositional flow and social physique anxiety in exercise setting?. Journal of Sports Science and Medicine.

[bib20] Guerin E., Bales E., Sweet S., Fortier M. (2012). A meta-analysis of the influence of gender on self-determination theory's motivational regulations for physical activity. Canadian Psychology-Psychologie Canadienne.

[bib21] Guerin E., Fortier M.S. (2012). Situational motivation and perceived intensity: Their interaction in predicting changes in positive affect from physical activity. J Obes.

[bib22] Gunnell K.E., Crocker P.R.E., Mack D.E., Wilson P.M., Zumbo B.D. (2014). Goal contents, motivation, psychological need satisfaction, well-being and physical activity: A test of self-determination theory over 6 months. Psychology of Sport and Exercise.

[bib23] Hallal P.C., Andersen L.B., Bull F.C., Guthold R., Haskell W., Ekelund U. (2012). Global physical activity levels: Surveillance progress, pitfalls, and prospects. The Lancet.

[bib24] Hamilton K., White K.M. (2010). Understanding parental physical activity: Meanings, habits, and social role influence. Psychology of Sport and Exercise.

[bib25] Henson J.M., Reise S.P., Kim K.H. (2007). Detecting mixtures from structural model differences using latent variable mixture modeling: A comparison of relative model fit statistics. Structural Equation Modeling-a Multidisciplinary Journal.

[bib26] Howard J., Gagne M., Bureau J.S. (2017). Testing a continuum structure of self-determined motivation: A meta-analysis. Psychological Bulletin.

[bib27] Howard J., Gagne M., Morin A.J.S., Van den Broeck A. (2016). Motivation profiles at work: A self-determination theory approach. Journal of Vocational Behavior.

[bib69] Hu L., Bentler P.M. (1999). Cutoff criteria for fit indexes in covariance structure analysis: Conventional criteria versus new alternatives. Structural Equation Modeling: A Multidisciplinary Journal.

[bib28] Hwang J., Kim Y.H. (2013). Physical activity and its related motivational attributes in adolescents with different BMI. International Journal of Behavioral Medicine.

[bib29] Jaakkola T., Wang C.K.J., Soini M., Liukkonen J. (2015). Students' perceptions of motivational climate and enjoyment in Finnish physical education: A latent profile Analysis. Journal of Sports Science and Medicine.

[bib65] Jago R., Salway R., Emm-Collison L., Sebire S., Thompson J., Lawlor D. (2019). Association of BMI category with change in children’s physical activity between ages 6 and 11 years: a longitudinal study. International Journal of Obesity.

[bib30] Jago R., Salway R., Lawlor D.A., Emm-Collison L., Heron J., Thompson J.L., Sebire S.J. (2018). Profiles of children's physical activity and sedentary behaviour between age 6 and 9: A latent profile and transition analysis. International Journal of Behavioral Nutrition and Physical Activity.

[bib66] Jago R., Sebire S., Wood L., Pool L., Zahra J., Thompson J., Lawlor D. (2014). Associations between objectively assessed child and parental physical activity: a cross-sectional study of families with 5-6 year old children. BMC Public Health.

[bib67] Jago R., Solomon-Moore E., Macdonald-Wallis C., Sebire S., Thompson J., Lawlor D. (2017). Change in children’s physical activity and sedentary time between Year 1 and Year 4 of primary school in the B-PROACT1V cohort. International Journal of Behavioral Nutrition and Physical Activity.

[bib68] Jago R., Thompson J., Sebire S., Wood L., Pool L., Zahra J., Lawlor D. (2014). Cross-sectional associations between the screen-time of parents and young children: differences by parent and child gender and day of the week. International Journal of Behavioral Nutrition and Physical Activity.

[bib31] Kapteyn A., Banks J., Hamer M., Smith J.P., Steptoe A., van Soest A., Wah S.H. (2018). What they say and what they do: Comparing physical activity across the USA, England and The Netherlands. Journal of Epidemiology & Community Health.

[bib32] Kay M.C., Carroll D.D., Carlson S.A., Fulton J.E. (2014). Awareness and knowledge of the 2008 physical activity guidelines for Americans. Journal of Physical Activity and Health.

[bib33] Kendzierski D. (1990). Exercise self-schemata - cognitive and behavioral-correlates. Health Psychology.

[bib34] Kyu H.H., Bachman V.F., Alexander L.T., Mumford J.E., Afshin A., Estep K., Forouzanfar M.H. (2016). Physical activity and risk of breast cancer, colon cancer, diabetes, ischemic heart disease, and ischemic stroke events: Systematic review and dose-response meta-analysis for the global burden of disease study 2013. BMJ.

[bib35] Lachman M.E., Lipsitz L., Lubben J., Castaneda-Sceppa C., Jette A.M. (2018). When adults don't exercise: Behavioral strategies to increase physical activity in sedentary middle-aged and older adults. Innov. Aging.

[bib36] LeBlanc A.G., Berry T., Deshpande S., Duggan M., Faulkner G., Latimer-Cheung A.E., Tremblay M.S. (2015). Knowledge and awareness of Canadian physical activity and sedentary behaviour guidelines: A synthesis of existing evidence. Applied Physiology Nutrition and Metabolism.

[bib37] Lindahl J., Stenling A., Lindwall M., Colliander C. (2015). Trends and knowledge base in sport and exercise psychology research: A bibliometric review study. International Review of Sport and Exercise Psychology.

[bib38] Lindwall M., Ivarsson A., Weman-Josefsson K., Jonsson L., Ntoumanis N., Patrick H., Teixeira P. (2017). Stirring the motivational soup: Within-person latent profiles of motivation in exercise. International Journal of Behavioral Nutrition and Physical Activity.

[bib39] Markland D., Tobin V. (2004). A modification to the behavioural regulation in exercise questionnaire to include an assessment of amotivation. Journal of Sport & Exercise Psychology.

[bib40] Martinent G., Decret J.C. (2015). Motivational profiles among young table-tennis players in intensive training settings: A latent profile transition analysis. Journal of Applied Sport Psychology.

[bib41] Mata J., Silva M.N., Vieira P.N., Carraca E.V., Andrade A.M., Coutinho S.R., Teixeira P.J. (2009). Motivational "Spill-Over" during weight control: Increased self-determination and exercise intrinsic motivation predict eating self-regulation. Health Psychology.

[bib42] Matsumoto H., Takenaka K. (2004). Motivational profiles and stages of exercise behavior change. International Journal of Sport and Health Science.

[bib43] McIntyre C.A., Rhodes R.E. (2009). Correlates of leisure-time physical activity during transitions to motherhood. Women & Health.

[bib44] Milyavskaya M., Koestner R. (2011). Psychological needs, motivation, and well-being: A test of self-determination theory across multiple domains. Personality and Individual Differences.

[bib45] Mokhtari S., Grace B., Pak Y., Reina A., Durand Q., Yee J.K. (2017). Motivation and perceived competence for healthy eating and exercise among overweight/obese adolescents in comparison to normal weight adolescents. BMC Obesity.

[bib46] Morin A.J.S., Meyer J.P., Creusier J., Bietry F. (2016). Multiple-group Analysis of similarity in latent profile solutions. Organizational Research Methods.

[bib47] Nadler J.T., Weston R., Voyles E.C. (2015). Stuck in the middle: The use and interpretation of mid-points in items on questionnaires. The Journal of General Psychology.

[bib48] Nylund-Gibson K., Grimm R., Quirk M., Furlong M. (2014). A latent transition mixture model using the three-step specification. Struc. Equ. Model.-a Multidiscip. J..

[bib49] Nylund K.L., Asparoutiov T., Muthen B.O. (2007). Deciding on the number of classes in latent class analysis and growth mixture modeling: A Monte Carlo simulation study. Structural Equation Modeling-a Multidisciplinary Journal.

[bib50] Office for National Statistics (2017). Estimated number of parents in families with dependent children by ethnic group of the parent. http://www.ons.gov.uk/peoplepopulationandcommunity.

[bib51] Prince S.A., Adamo K.B., Hamel M.E., Hardt J., Gorber S.C., Tremblay M. (2008). A comparison of direct versus self-report measures for assessing physical activity in adults: A systematic review. International Journal of Behavioral Nutrition and Physical Activity.

[bib52] Rebar A.L., Stanton R., Geard D., Short C., Duncan M.J., Vandelanotte C. (2015). A meta-meta-analysis of the effect of physical activity on depression and anxiety in non-clinical adult populations. Health Psychology Review.

[bib71] Ryan R.M., Deci E.L. (2000). The Darker and Brighter Sides of Human Existence: Basic Psychological Needs as a Unifying Concept. Psychological Inquiry.

[bib53] Ryan R.M., Deci E. (2017). Self-determination theory: Basic psychological needs in motivation, development, and wellness.

[bib54] Samdal G.B., Eide G.E., Barth T., Williams G., Meland E. (2017). Effective behaviour change techniques for physical activity and healthy eating in overweight and obese adults; systematic review and meta-regression analyses. International Journal of Behavioral Nutrition and Physical Activity.

[bib55] Sebire S.J., Toumpakari Z., Turner K.M., Cooper A.R., Page A.S., Malpass A., Andrews R.C. (2018). I've made this my lifestyle now": A prospective qualitative study of motivation for lifestyle change among people with newly diagnosed type two diabetes mellitus. BMC Public Health.

[bib56] Solomon-Moore E., Sebire S.J., Thompson J.L., Zahra J., Lawlor D.A., Jago R. (2016). Are parents' motivations to exercise and intention to engage in regular family-based activity associated with both adult and child physical activity?. BMJ Open Sport Exerc. Med..

[bib57] Standage M., Sebire S.J., Loney T. (2008). Does exercise motivation predict engagement in objectively assessed bouts of moderate-intensity exercise?: A self-determination theory perspective. Journal of Sport & Exercise Psychology.

[bib58] Tabachnick B.G., Fidell L.S., Tabachnick B.G., Fidell L.S. (2013). Cleaning up your act: Screening data prior to analysis. Using multivariate statistics.

[bib59] Troiano R.P., Berrigan D., Dodd K.W., Masse L.C., Tilert T., McDowell M. (2008). Physical activity in the United States measured by accelerometer. Medicine & Science in Sports & Exercise.

[bib60] Tudor-Locke C., Burkett L., Reis J.P., Ainsworth B.E., Macera C.A., Wilson D.K. (2005). How many days of pedometer monitoring predict weekly physical activity in adults?. Preventive Medicine.

[bib61] US Department of Health and Human Services (2018). Physical activity guidelines for Americans.

[bib62] Vansteenkiste M., Mouratidis A. (2016). Emerging trends and future directions for the field of motivation psychology: A special issue in honor of prof. Dr. Willy lens. Psychologica Belgica.

[bib63] Weman-Josefsson K., Lindwall M., Ivarsson A. (2015). Need satisfaction, motivational regulations and exercise: Moderation and mediation effects. International Journal of Behavioral Nutrition and Physical Activity.

[bib64] Yang C.C. (2006). Evaluating latent class analysis models in qualitative phenotype identification. Computational Statistics & Data Analysis.

