# Supplementary material for: Multidimensional motivation for exercise: A latent profile and transition analysis
Source: Psychol Sport Exerc. 2020 Mar;47:101619. doi: 10.1016/j.psychsport.2019.101619 (PMC7015274; doi:10.1016/j.psychsport.2019.101619)
Supplement: Multimedia component [file mmc1.docx]

**Table S1**

*Correlation Matrix of all Variables in the Study*

|  | **1** | **2** | **3** | **4** | **5** | **6** | **7** | **8** | **9** | **10** | **11** | **12** | **13** | **14** | **15** | **16** | **17** | **18** | **19** | **20** | **21** | **22** |
| --- | --- | --- | --- | --- | --- | --- | --- | --- | --- | --- | --- | --- | --- | --- | --- | --- | --- | --- | --- | --- | --- | --- |
| 1. Gender | - |  |  |  |  |  |  |  |  |  |  |  |  |  |  |  |  |  |  |  |  |  |
| 2.Baseline age | -0.04 | - |  |  |  |  |  |  |  |  |  |  |  |  |  |  |  |  |  |  |  |  |
| **Time 1** | | | | | | | | | | | | | | | | | | | | | | |
| 1. 3. BMI | -0.04 | 0.16 | - |  |  |  |  |  |  |  |  |  |  |  |  |  |  |  |  |  |  |  |
| 1. 4. MVPA | 0.01 | 0.12 | 0.06 | - |  |  |  |  |  |  |  |  |  |  |  |  |  |  |  |  |  |  |
| 1. 5. Intrinsic | -0.09 | 0.02 | 0.10 | 0.04 | - |  |  |  |  |  |  |  |  |  |  |  |  |  |  |  |  |  |
| 1. 6. Identified | -0.07 | 0.04 | 0.10 | 0.04 | 0.94 | - |  |  |  |  |  |  |  |  |  |  |  |  |  |  |  |  |
| 1. 7. Introjected | 0.01 | -0.02 | 0.05 | 0.01 | 0.30 | 0.46 | - |  |  |  |  |  |  |  |  |  |  |  |  |  |  |  |
| 1. 8. Extrinsic | 0.01 | -0.01 | 0.00 | -0.03 | -0.19 | -0.14 | 0.43 | - |  |  |  |  |  |  |  |  |  |  |  |  |  |  |
| 1. 9. Amotivation | 0.02 | -0.03 | -0.07 | -0.05 | -0.57 | -0.64 | -0.13 | 0.42 | - |  |  |  |  |  |  |  |  |  |  |  |  |  |
| **Time 2** | | | | | | | | | | | | | | | | | | | | | | |
| 1. 10. BMI | -0.06 | 0.02 | 0.15 | 0.00 | 0.02 | 0.04 | 0.09 | 0.05 | -0.07 |  |  |  |  |  |  |  |  |  |  |  |  |  |
| 1. 11. MVPA | 0.03 | 0.02 | 0.09 | 0.05 | 0.11 | 0.12 | 0.06 | -0.01 | -0.08 | 0.11 | - |  |  |  |  |  |  |  |  |  |  |  |
| 1. 12. Intrinsic | 0.02 | 0.03 | 0.07 | 0.02 | 0.57 | 0.56 | 0.17 | -0.15 | -0.35 | 0.03 | 0.06 | - |  |  |  |  |  |  |  |  |  |  |
| 1. 13. Identified | -0.03 | 0.04 | 0.08 | 0.01 | 0.57 | 0.61 | 0.26 | -0.09 | -0.36 | 0.04 | 0.05 | 0.90 | - |  |  |  |  |  |  |  |  |  |
| 1. 14. Introjected | -0.05 | -0.01 | 0.04 | -0.02 | 0.20 | 0.28 | 0.45 | 0.20 | -0.07 | 0.06 | -0.07 | 0.25 | 0.47 | - |  |  |  |  |  |  |  |  |
| 1. 15. Extrinsic | -0.05 | 0.06 | -0.02 | 0.01 | -0.14 | -0.10 | 0.13 | 0.25 | 0.14 | -0.04 | -0.03 | -0.27 | -0.19 | 0.35 | - |  |  |  |  |  |  |  |
| 1. 16. Amotivation | -0.02 | 0.02 | -0.06 | 0.02 | -0.39 | -0.41 | -0.12 | 0.14 | 0.33 | -0.03 | -0.03 | -0.55 | -0.63 | -0.04 | 0.50 | - |  |  |  |  |  |  |
| **Time 3** | | | | | | | | | | | | | | | | | | | | | | |
| 1. 17. BMI | 0.05 | 0.03 | -0.04 | 0.06 | 0.06 | 0.07 | 0.02 | 0.01 | -0.05 | 0.00 | 0.06 | -0.04 | -0.02 | 0.00 | 0.06 | 0.01 | - |  |  |  |  |  |
| 1. 18. MVPA | -0.04 | 0.08 | 0.09 | 0.13 | -0.05 | -0.04 | 0.09 | 0.06 | 0.06 | -0.03 | -0.04 | -0.03 | 0.00 | 0.07 | 0.09 | 0.05 | 0.18 | - |  |  |  |  |
| 1. 19. Intrinsic | -0.03 | -0.05 | -0.02 | 0.06 | 0.52 | 0.50 | 0.15 | -0.15 | -0.28 | -0.08 | 0.02 | 0.59 | 0.55 | 0.08 | -0.20 | -0.36 | -0.03 | -0.05 | - |  |  |  |
| 1. 20. Identified | -0.08 | -0.05 | -0.02 | 0.06 | 0.52 | 0.56 | 0.25 | -0.09 | -0.30 | -0.07 | 0.00 | 0.56 | 0.61 | 0.20 | -0.15 | -0.39 | 0.00 | -0.02 | 0.90 | - |  |  |
| 1. 21. Introjected | -0.11 | 0.02 | 0.02 | 0.01 | 0.21 | 0.29 | 0.47 | 0.18 | -0.15 | 0.00 | -0.04 | 0.18 | 0.28 | 0.44 | 0.16 | -0.08 | 0.05 | 0.10 | 0.25 | 0.47 | - |  |
| 1. 22. Extrinsic | -0.08 | 0.06 | 0.04 | -0.05 | -0.13 | -0.09 | 0.19 | 0.30 | 0.10 | 0.09 | -0.05 | -0.15 | -0.14 | 0.20 | 0.34 | 0.19 | -0.02 | 0.09 | -0.24 | -0.19 | 0.37 | - |
| 1. 23. Amotivation | 0.02 | 0.06 | 0.02 | -0.04 | -0.35 | -0.37 | -0.11 | 0.12 | 0.30 | 0.02 | -0.02 | -0.40 | -0.44 | -0.10 | 0.19 | 0.48 | -0.01 | 0.06 | -0.57 | -0.65 | -0.16 | 0.45 |

**Table S2**

*Factor Score Determinacy Coefficients*

|  | Time 1 | Time 2 | Time 3 |
| --- | --- | --- | --- |
| Intrinsic regulation | .97 | .97 | .97 |
| Identified regulation | .95 | .95 | .96 |
| Introjected regulation | .89 | .91 | .91 |
| Extrinsic regulation | .86 | .91 | .89 |
| Amotivation | .89 | .92 | .92 |

**Table S3**

*Model fit Indices from Longitudinal Invariance Testing of the CFA model*

| Model | χ^2^ | df | p | CFI | RMSEA | 90% CI |  | SRMR |
| --- | --- | --- | --- | --- | --- | --- | --- | --- |
| Configural | 2803.87 | 1380 | 0.000 | .95 | .02 | .02, .03 |  | .05 |
| Metric | 2819.67 | 1408 | 0.000 | .95 | .02 | .02, .02 |  | .05 |
| Scalar | 3163.32 | 1428 | 0.000 | .94 | .03 | .02, .03 |  | .06 |
| Residual | 330.32 | 1461 | 0.000 | .94 | .03 | .03, .03 |  | .06 |

**Table S4**

*Model Fit Indices for ESEM*

|  | χ^2^ | df | p | CFI | RMSEA | 90% CI | SRMR |
| --- | --- | --- | --- | --- | --- | --- | --- |
| Time 1 | 335.21 | 86 | 0.00 | .96 | .05 | .05, .06 | .02 |
| Time 2 | 214.75 | 86 | 0.00 | .98 | .04 | .03, .05 | .01 |
| Time 3 | 190.53 | 86 | 0.00 | .99 | .03 | .03, .04 | .01 |

**Table S5**

*Profiles With Correlated Indicators*

|  | Profile 1 | Profile 2 | Profile 3 | Profile 4 | Profile 5 | Profile 6 |
| --- | --- | --- | --- | --- | --- | --- |
| Intrinsic regulation | -1.29 | -1.10 | -0.35 | 0.09 | 0.74 | 0.56 |
| Identified regulation | -1.16 | -1.02 | -0.11 | 0.13 | 0.73 | 0.38 |
| Introjected regulation | -0.26 | -0.62 | 0.81 | -0.07 | 1.23 | -0.12 |
| Extrinsic regulation | 0.45 | -0.06 | 1.24 | -0.05 | -0.02 | -0.29 |
| Amotivation | 1.31 | 0.14 | 0.31 | -0.12 | -0.20 | -0.26 |

**Table S6**

*Profiles With no Model Constraints*

|  | Profile 1 | Profile 2 | Profile 3 | Profile 4 | Profile 5 | Profile 6 |
| --- | --- | --- | --- | --- | --- | --- |
| **Time 1** |  |  |  |  |  |  |
| Intrinsic regulation | -2.07 | -1.27 | -0.72 | -0.15 | 0.03 | 0.93 |
| Identified regulation | -1.80 | -1.05 | -0.59 | -0.11 | 0.19 | 0.73 |
| Introjected regulation | -0.61 | -0.61 | 0.41 | -0.19 | 0.98 | 0.25 |
| Extrinsic regulation | 0.20 | -0.08 | 0.90 | -0.13 | 0.89 | -0.15 |
| Amotivation | 1.81 | 0.27 | 1.08 | -0.14 | 0.00 | -0.28 |
| **Time 2** |  |  |  |  |  |  |
| Intrinsic regulation | -1.73 | -1.42 | -0.70 | -0.11 | -0.23 | 0.94 |
| Identified regulation | -1.58 | -1.06 | -0.62 | -0.10 | 0.11 | 0.71 |
| Introjected regulation | -0.59 | -0.60 | 0.45 | -0.23 | 0.76 | 0.25 |
| Extrinsic regulation | 0.12 | -0.08 | 1.21 | -0.18 | 1.07 | -0.24 |
| Amotivation | 1.33 | 0.11 | 1.10 | -0.14 | 0.08 | -0.28 |
| **Time 3** |  |  |  |  |  |  |
| Intrinsic regulation | -1.32 | -1.69 | -0.90 | -0.34 | -0.17 | 0.85 |
| Identified regulation | -1.23 | -1.29 | -0.81 | -0.28 | 0.03 | 0.67 |
| Introjected regulation | -0.59 | -0.78 | 0.38 | -0.37 | 0.74 | 0.28 |
| Extrinsic regulation | 0.18 | -0.15 | 1.66 | -0.19 | 0.93 | -0.21 |
| Amotivation | 1.10 | 0.07 | 1.59 | -0.14 | 0.17 | -0.28 |

**MPLus Input for Step 1 and 2**

*! This code is an example of the process outlined in step 1. This shows the input file when estimating 6 profiles but we additionally estimated for 2 to 7 profiles.*

DATA:

FILE IS FSCORES.dat;

VARIABLE:

NAMES ARE school use sed_3 sed_2 sed_1 mvpa_3 mvpa_2 mvpa_1

match_2_3 match_1_3 match_1_2 light_3 light_2 light_1 gender_3

gender_2 gender_1 ethnic_3 ethnic_2 ethnic_1 cpm_3 cpm_2 cpm_1

intro_av_3 intro_av_2 intro_av_1 intri_av_3 intri_av_2

intri_av_1 iden_av_3 iden_av_2 iden_av_1 ext_av_3

ext_av_2 ext_av_1 amot_av_3 amot_av_2 amot_av_1 bmi_3 bmi_2

bmi_1 age_3 age_2 age_1 acc_valid_data_3 acc_valid_data_2

acc_valid_data_1 imd_3 imd_2 imd_1 id intri_1 iden_1 intro_1

ext_1 amot_1 intri_2 iden_2 intro_2 ext_2 amot_2 intri_3 iden_3

intro_3 ext_3 amot_3;

IDVARIABLE=id;

MISSING ARE ALL (-9999);

*! To estimate a different number of profiles replace as follows: CLASSES=C1(N) C2(N) C3(N);*

CLASSES=C1(6) C2(6) C3(6);

USEVARIABLES= intri_1 iden_1 intro_1 ext_1 amot_1 intri_2

iden_2 intro_2 ext_2 amot_2 intri_3 iden_3 intro_3 ext_3 amot_3;

CLUSTER=school;

ANALYSIS:

TYPE=mixture missing complex;

ESTIMATOR=ML;

*! The model was also estimated using 1000 200 starts to ensure the outcome was stable*

STARTS=500 100;

MODEL:

MODEL C1:

%C1#1%

[intri_1-amot_1] (v11-v15);

%C1#2%

[intri_1-amot_1] (v16-v20);

%C1#3%

[intri_1-amot_1] (v21-v25);

%C1#4%

[intri_1-amot_1] (v26-v30);

%C1#5%

[intri_1-amot_1] (v31-v35);

%C1#6%

[intri_1-amot_1] (v36-v40);

MODEL C2:

%C2#1%

[intri_2-amot_2] (v11-v15);

%C2#2%

[intri_2-amot_2] (v16-v20);

%C2#3%

[intri_2-amot_2] (v21-v25);

%C2#4%

[intri_2-amot_2] (v26-v30);

%C2#5%

[intri_2-amot_2] (v31-v35);

%C2#6%

[intri_2-amot_2] (v36-v40);

MODEL C3:

%C3#1%

[intri_3-amot_3] (v11-v15);

%C3#2%

[intri_3-amot_3] (v16-v20);

%C3#3%

[intri_3-amot_3] (v21-v25);

%C3#4%

[intri_3-amot_3] (v26-v30);

%C3#5%

[intri_3-amot_3] (v31-v35);

%C3#6%

[intri_3-amot_3] (v36-v40);

OUTPUT: svalues TECH14;

SAVEDATA:

FILE is 6c_cprob.dat;

**MPLus Input for Step 3**

DATA:

FILE IS "C36CLASS";

VARIABLE:

NAMES ARE use intri_3 iden_3 intro_3 ext_3 amot_3 id intri_2 iden_2 intro_2 ext_2 amot_2 intri_1 iden_1 intro_1 ext_1 amot_1 gender_1 age_1 bmi_1 mvpa_1 light_1 sed_1 gender_2 age_2 bmi_2 mvpa_2 light_2 sed_2 gender_3 age_3 bmi_3 mvpa_3 light_3 sed_3 c1prob1 c1prob2 c1prob3 c1prob4 c1prob5 c1prob6 n1 c2prob1 c2prob2 c2prob3 c2prob4 c2prob5 c2prob6 n2 c3prob1 c3prob2 c3prob3 c3prob4 c3prob5 c3prob6 n3 school ;

IDVARIABLE=id;

MISSING ARE ALL(-9999);

CLASSES=C1 (6) C2 (6) C3(6);

USEVARIABLES= N1 N2 N3;

NOMINAL= N1 N2 N3;

ANALYSIS:

TYPE=mixture missing complex;

STARTS=500 100;

MODEL:

%OVERALL%

C2 ON C1;

C3 ON C2;

MODEL C1:

%C1#1%

[N1#1@3.742]; [N1#2@-4.492]; [N1#3@-0.302]; [N1#4@-1.912]; [N1#5@-1.431];

*! values are derived from step 1 and 2 analyses*

%C1#2%

[N1#1@13.106]; [N1#2@13.070]; [N1#3@10.208]; [N1#4@8.807]; [N1#5@0.000];

%C1#3%

[N1#1@13.197]; [N1#2@9.102]; [N1#3@13.017]; [N1#4@7.724]; [N1#5@0.000];

%C1#4%

[N1#1@10.810]; [N1#2@6.092]; [N1#3@6.177]; [N1#4@10.731]; [N1#5@5.427];

%C1#5%

[N1#1@2.088]; [N1#2@-11.070]; [N1#3@-11.070]; [N1#4@-2.461]; [N1#5@1.860];

%C1#6%

[N1#1@0.165]; [N1#2@-13.009]; [N1#3@-13.009]; [N1#4@-9.694]; [N1#5@-2.812];

MODEL C2:

%C2#1%

[N2#1@-0.281]; [N2#2@-13.230]; [N2#3@-3.786]; [N2#4@-4.836]; [N2#5@-4.571];

%C2#2%

[N2#1@-3.520]; [N2#2@-0.150]; [N2#3@-3.715]; [N2#4@-4.806]; [N2#5@-13.162];

%C2#3%

[N2#1@-2.899]; [N2#2@-4.267]; [N2#3@-0.248]; [N2#4@-4.712]; [N2#5@-13.196];

%C2#4%

[N2#1@-3.450]; [N2#2@-4.506]; [N2#3@-4.856]; [N2#4@-0.188]; [N2#5@-5.238];

%C2#5%

[N2#1@-3.749]; [N2#2@-13.318]; [N2#3@-13.318]; [N2#4@-5.351]; [N2#5@-0.484];

%C2#6%

[N2#1@-3.358]; [N2#2@-13.756]; [N2#3@-13.756]; [N2#4@-7.586]; [N2#5@-3.651];

MODEL C3:

%C3#1%

[N3#1@3.304]; [N3#2@-8.593]; [N3#3@-1.044]; [N3#4@-1.719]; [N3#5@-1.468];

%C3#2%

[N3#1@13.1284]; [N3#2@13.031]; [N3#3@10.344]; [N3#4@9.211]; [N3#5@0.000];

%C3#3%

[N3#1@4.161]; [N3#2@0.135]; [N3#3@3.895]; [N3#4@-0.263]; [N3#5@-9.060];

%C3#4%

[N3#1@8.540]; [N3#2@4.659]; [N3#3@4.716]; [N3#4@8.390]; [N3#5@4.262];

%C3#5%

[N3#1@2.041]; [N3#2@-11.092]; [N3#3@-11.092]; [N3#4@-2.669]; [N3#5@1.866];

%C3#6%

[N3#1@0.183]; [N3#2@-12.993]; [N3#3@-12.993]; [N3#4@-7.351]; [N3#5@-2.588];

**MPLus Input for Step 4- Gender**

*! The input files show estimation with time 3 data but were also run using time 1 and time 2 data.*

DATA:

FILE IS "F:\LPA and transition analysis\Alltimepoints.csv";

VARIABLE:

NAMES ARE school use sed_3 sed_2 sed_1 mvpa_3 mvpa_2 mvpa_1

match_2_3 match_1_3 match_1_2 light_3 light_2 light_1 gender_3

gender_2 gender_1 ethnic_3 ethnic_2 ethnic_1 cpm_3 cpm_2 cpm_1

intro_av_3 intro_av_2 intro_av_1 intri_av_3 intri_av_2

intri_av_1 iden_av_3 iden_av_2 iden_av_1 ext_av_3

ext_av_2 ext_av_1 amot_av_3 amot_av_2 amot_av_1 bmi_3 bmi_2

bmi_1 age_3 age_2 age_1 acc_valid_data_3 acc_valid_data_2

acc_valid_data_1 imd_3 imd_2 imd_1 id intri_1 iden_1 intro_1

ext_1 amot_1 intri_2 iden_2 intro_2 ext_2 amot_2 intri_3 iden_3

intro_3 ext_3 amot_3;

IDVARIABLE=id;

MISSING ARE ALL (-9999);

CLASSES= C1(6);

USEVARIABLES= intri_3 iden_3 intro_3 ext_3 amot_3 use;

AUXILIARY= intri_1 iden_1 intro_1 ext_1

amot_1 intri_2 iden_2 intro_2 ext_2 amot_2 gender_3 (dcategorical) age_1 bmi_1 mvpa_1

light_1 sed_1 gender_2 age_2 bmi_2 mvpa_2 light_2 sed_2 gender_1 age_3

bmi_3 mvpa_3 light_3 sed_3;

!AUXILLIARY= gender_3 (BCH);

CATEGORICAL ARE use;

ANALYSIS:

TYPE=mixture missing;

MODEL:

*! this stage uses values identified through step 1 and 2*

%C1#1%

[intri_3@-0.27260 ] (m21); [iden_3@-0.21827 ] (m22); [intro_3@-0.27994 ] (m23);

[ext_3@-0.11420 ] (m24); [amot_3@-0.11550 ] (m25);

%C1#2%

[intri_3@-1.68499 ] (m11); [iden_3@-1.51577 ] (m12); [intro_3@-0.55526 ] (m13);

[ext_3@0.25650 ] (m14); [amot_3@1.44182 ] (m15);

%C1#3%

[intri_3@-1.44573 ] (m16); [iden_3@-1.15006 ] (m17); [intro_3@-0.67593 ] (m18);

[ext_3@-0.08468 ] (m19); [amot_3@0.20189 ] (m20);

%C1#4%

[intri_3@-0.53493 ] (m36); [iden_3@-0.34917 ] (m37); [intro_3@0.63229 ] (m38);

[ext_3@1.09393 ] (m39); [amot_3@0.70790 ] (m40);

%C1#5%

[intri_3@0.69333 ] (m31); [iden_3@0.73420 ] (m32); [intro_3@1.13879 ] (m33);

[ext_3@0.09813 ] (m34); [amot_3@-0.23155 ] (m35);

%C1#6%

[intri_3@0.89496 ] (m26); [iden_3@0.61853 ] (m27); [intro_3@-0.15933 ] (m28);

[ext_3@-0.24102 ] (m29); [amot_3@-0.27691 ] (m30);

**MPLus Input for Step 4- BMI**

DATA:

FILE IS "F:\LPA and transition analysis\Alltimepoints.csv";

VARIABLE:

NAMES ARE school use sed_3 sed_2 sed_1 mvpa_3 mvpa_2 mvpa_1

match_2_3 match_1_3 match_1_2 light_3 light_2 light_1 gender_3

gender_2 gender_1 ethnic_3 ethnic_2 ethnic_1 cpm_3 cpm_2 cpm_1

intro_av_3 intro_av_2 intro_av_1 intri_av_3 intri_av_2

intri_av_1 iden_av_3 iden_av_2 iden_av_1 ext_av_3

ext_av_2 ext_av_1 amot_av_3 amot_av_2 amot_av_1 bmi_3 bmi_2

bmi_1 age_3 age_2 age_1 acc_valid_data_3 acc_valid_data_2

acc_valid_data_1 imd_3 imd_2 imd_1 id intri_1 iden_1 intro_1

ext_1 amot_1 intri_2 iden_2 intro_2 ext_2 amot_2 intri_3 iden_3

intro_3 ext_3 amot_3;

IDVARIABLE=id;

MISSING ARE ALL (-9999);

CLASSES= C1(6);

USEVARIABLES= intri_3 iden_3 intro_3 ext_3 amot_3 use;

AUXILIARY= intri_1 iden_1 intro_1 ext_1

amot_1 intri_2 iden_2 intro_2 ext_2 amot_2 gender_1 age_1 bmi_3 (dcontinuous) mvpa_1

light_1 sed_1 gender_2 age_2 bmi_1 mvpa_2 light_2 sed_2 gender_3 age_3

bmi_2 mvpa_3 light_3 sed_3;

!AUXILLIARY= bmi_3 (BCH);

CATEGORICAL ARE use;

ANALYSIS:

TYPE=mixture missing;

MODEL:

%C1#1%

[intri_3@-0.27260 ] (m21); [iden_3@-0.21827 ] (m22); [intro_3@-0.27994 ] (m23);

[ext_3@-0.11420 ] (m24); [amot_3@-0.11550 ] (m25);

%C1#2%

[intri_3@-1.68499 ] (m11); [iden_3@-1.51577 ] (m12); [intro_3@-0.55526 ] (m13);

[ext_3@0.25650 ] (m14); [amot_3@1.44182 ] (m15);

%C1#3%

[intri_3@-1.44573 ] (m16); [iden_3@-1.15006 ] (m17); [intro_3@-0.67593 ] (m18);

[ext_3@-0.08468 ] (m19); [amot_3@0.20189 ] (m20);

%C1#4%

[intri_3@-0.53493 ] (m36); [iden_3@-0.34917 ] (m37); [intro_3@0.63229 ] (m38);

[ext_3@1.09393 ] (m39); [amot_3@0.70790 ] (m40);

%C1#5%

[intri_3@0.69333 ] (m31); [iden_3@0.73420 ] (m32); [intro_3@1.13879 ] (m33);

[ext_3@0.09813 ] (m34); [amot_3@-0.23155 ] (m35);

%C1#6%

[intri_3@0.89496 ] (m26); [iden_3@0.61853 ] (m27); [intro_3@-0.15933 ] (m28);

[ext_3@-0.24102 ] (m29); [amot_3@-0.27691 ] (m30);

**MPlus Input for Step 4- MVPA**

DATA:

FILE IS "F:\LPA and transition analysis\Alltimepoints.csv";

VARIABLE:

NAMES ARE school use sed_3 sed_2 sed_1 mvpa_3 mvpa_2 mvpa_1

match_2_3 match_1_3 match_1_2 light_3 light_2 light_1 gender_3

gender_2 gender_1 ethnic_3 ethnic_2 ethnic_1 cpm_3 cpm_2 cpm_1

intro_av_3 intro_av_2 intro_av_1 intri_av_3 intri_av_2

intri_av_1 iden_av_3 iden_av_2 iden_av_1 ext_av_3

ext_av_2 ext_av_1 amot_av_3 amot_av_2 amot_av_1 bmi_3 bmi_2

bmi_1 age_3 age_2 age_1 acc_valid_data_3 acc_valid_data_2

acc_valid_data_1 imd_3 imd_2 imd_1 id intri_1 iden_1 intro_1

ext_1 amot_1 intri_2 iden_2 intro_2 ext_2 amot_2 intri_3 iden_3

intro_3 ext_3 amot_3;

IDVARIABLE=id;

MISSING ARE ALL (-9999);

CLASSES= C3(6);

USEVARIABLES= intri_3 iden_3 intro_3 ext_3 amot_3 use;

AUXILIARY= intri_1 iden_1 intro_1 ext_1

amot_1 intri_2 iden_2 intro_2 ext_2 amot_2 gender_1 age_1 bmi_1 mvpa_1

light_1 sed_1 gender_2 age_2 bmi_2 mvpa_2 light_2 sed_2 gender_3 age_3

bmi_3 mvpa_3 (dcontinuous) light_3 sed_3;

!AUXILLIARY= mvpa_3 (BCH);

CATEGORICAL ARE use;

ANALYSIS:

TYPE=mixture missing;

MODEL:

%C3#1%

[intri_3@-0.27260 ] (m21); [iden_3@-0.21827 ] (m22); [intro_3@-0.27994 ] (m23);

[ext_3@-0.11420 ] (m24); [amot_3@-0.11550 ] (m25);

%C3#2%

[intri_3@-1.68499 ] (m11); [iden_3@-1.51577 ] (m12); [intro_3@-0.55526 ] (m13);

[ext_3@0.25650 ] (m14); [amot_3@1.44182 ] (m15);

%C3#3%

[intri_3@-1.44573 ] (m16); [iden_3@-1.15006 ] (m17); [intro_3@-0.67593 ] (m18);

[ext_3@-0.08468 ] (m19); [amot_3@0.20189 ] (m20);

%C3#4%

[intri_3@-0.53493 ] (m36); [iden_3@-0.34917 ] (m37); [intro_3@0.63229 ] (m38);

[ext_3@1.09393 ] (m39); [amot_3@0.70790 ] (m40);

%C3#5%

[intri_3@0.69333 ] (m31); [iden_3@0.73420 ] (m32); [intro_3@1.13879 ] (m33);

[ext_3@0.09813 ] (m34); [amot_3@-0.23155 ] (m35);

%C3#6%

[intri_3@0.89496 ] (m26); [iden_3@0.61853 ] (m27); [intro_3@-0.15933 ] (m28);

[ext_3@-0.24102 ] (m29); [amot_3@-0.27691 ] (m30);
